# Supplementary material for: Signatures of Dermal Fibroblasts from RDEB Pediatric Patients
Source: Int J Mol Sci. 2021 Feb 11;22(4):1792. doi: 10.3390/ijms22041792 (PMC7918539; doi:10.3390/ijms22041792)
Supplement: Supplementary file 1 [file ijms-22-01792-s001.zip › ijms-1080037-proofed-supplementary/Supplementary Discussion-edited.docx]

**3S. Supplementary Discussion.**

Here, we briefly describe the differential expression of several genes in the FEB lines.

In FEB2 and FEB3, we found the reduced level of the DSP transcript, which encodes an essential component of functional desmosomes. Change in DSP expression has not been shown previously for RDEB. However, the analyzed transcriptomes demonstrated an elevated (FC > 5) level of DSP mRNA in RDEB patients' fibroblasts with c.6527insC relative to their controls (Supplementary Table S3). The upregulation of DSP was shown in the model of airway fibrosis upon treatment with TGF-β, where enhanced expression of DSP was directly associated with the myofibroblast phenotype [1]. Thus, the decrease of DSP mRNA in our FEB lines could be noteworthy, as the profibrotic expression of these lines appears to be nontypical.

In spite of the fact that alpha-smooth muscle actin (α-SMA) expression was enhanced in all the FEB lines (Figure 3B,B), we detected the upregulation of α-SMA mRNA only in the FEB4 and FEB3 lines (Supplementary Figure S7). These lines demonstrated the similar enhanced expression trend for the CCN2-encoding gene. CCN2 is a matricellular protein attributed to myofibroblasts and has been shown to possess typical profibrotic traits. All FEB lines also demonstrated decreased expression of the signaling inhibitor SMAD7, which regulates both TGF-β-dependent and BMP-induced SMAD signaling [2].

The decreased expression of COL4A1 mRNA was found in all FEB lines except FEB3 (Supplementary Figure S7). It is possible that the thinning of the basal membrane (BM) observed in RDEB could be the consequence of COL4A1 downregulation [4]. Proteome studies confirm a decrease in the expression of laminin-332 and type IV collagen as the main components of basal membrane due to the loss of C7 [4,5]. These findings support the hypothesis of age-associated changes in the skin of RDEB patients [6].

Glycoproteins are involved in the organization of the extracellular matrix and cellular signaling. Although GLDN is not directly involved in fibrosis, its increase could lead to an enhancement of ECM protein content. The increased expression of GLDN in FEB lines was demonstrated (Supplementary Figure S7).

STEAP4, together with MELTF, which is also found to be differential in some of the FEBs (Figure 5B), belong to the GO group of ion homeostasis, both of them mediating iron-associated homeostasis. MELTF is involved in iron uptake, and the upregulated expression of MELTF correlates with the development of some types of cancers [7]. Interestingly, the FEB with a less severe phenotype was associated with a higher expression level of the MELTF transcript. As both an excess and a lack of iron is adverse for cells, the mechanism regulating iron uptake might be of tight control.

1[. Walker, E.J.; Heydet, D.; Veldre, T.; Ghildyal, R. Transcriptomic Changes during TGF-β-Mediated Differentiation of Airway Fibroblasts to Myofibroblasts.](https://www.zotero.org/google-docs/?t7TI80) *Sci. Rep.* **2019**, *9*, 20377, doi:10.1038/s41598-019-56955-1.

2[. Miyazawa, K.; Miyazono, K. Regulation of TGF-β Family Signaling by Inhibitory Smads. *Cold Spring Harb. Perspect. Biol.* **2017**, *9*, doi:10.1101/cshperspect.a022095.](https://www.zotero.org/google-docs/?XGHUF8)

3[. Schindelin, J.; Arganda-Carreras, I.; Frise, E.; Kaynig, V.; Longair, M.; Pietzsch, T.; Preibisch, S.; Rueden, C.; Saalfeld, S.; Schmid, B.; et al. Fiji: An Open-Source Platform for Biological-Image Analysis. *Nat. Methods* **2012**, *9*, 676–682, doi:10.1038/nmeth.2019.](https://www.zotero.org/google-docs/?t7TI80)

4[. Thriene, K.; Grüning, B.A.; Bornert, O.; Erxleben, A.; Leppert, J.; Athanasiou, I.; Weber, E.; Kiritsi, D.; Nyström, A.; Reinheckel, T.; et al. Combinatorial Omics Analysis Reveals Perturbed Lysosomal Homeostasis in Collagen VII-Deficient Keratinocytes. *Mol. Cell. Proteomics MCP* **2018**, *17*, 565–579, doi:10.1074/mcp.RA117.000437.](https://www.zotero.org/google-docs/?t7TI80)

5[. Küttner, V.; Mack, C.; Rigbolt, K.T.; Kern, J.S.; Schilling, O.; Busch, H.; Bruckner-Tuderman, L.; Dengjel, J. Global Remodelling of Cellular Microenvironment Due to Loss of Collagen VII. *Mol. Syst. Biol.* **2013**, *9*, 657, doi:10.1038/msb.2013.17.](https://www.zotero.org/google-docs/?t7TI80)

6[. Breitenbach, J.S.; Rinnerthaler, M.; Trost, A.; Weber, M.; Klausegger, A.; Gruber, C.; Bruckner, D.; Reitsamer, H.A.; Bauer, J.W.; Breitenbach, M. Transcriptome and Ultrastructural Changes in Dystrophic Epidermolysis Bullosa Resemble Skin Aging. *Aging* **2015**, *7*, 389–411, doi:10.18632/aging.100755.](https://www.zotero.org/google-docs/?t7TI80)

7[. Lane, D.J.R.; Merlot, A.M.; Huang, M.L.-H.; Bae, D.-H.; Jansson, P.J.; Sahni, S.; Kalinowski, D.S.; Richardson, D.R. Cellular Iron Uptake, Trafficking and Metabolism: Key Molecules and Mechanisms and Their Roles in Disease. *Biochim. Biophys. Acta BBA - Mol. Cell Res.* **2015**, *1853*, 1130–1144, doi:10.1016/j.bbamcr.2015.01.021.](https://www.zotero.org/google-docs/?t7TI80)
