# Supplementary material for: Signatures of Dermal Fibroblasts from RDEB Pediatric Patients
Source: Int J Mol Sci. 2021 Feb 11;22(4):1792. doi: 10.3390/ijms22041792 (PMC7918539; doi:10.3390/ijms22041792)
Supplement: Supplementary file 1 [file ijms-22-01792-s001.zip › ijms-1080037-proofed-supplementary/Supplementary Methods-edited.docx]

**Supplementary Methods**

**1. List of Primers**

All synthetic DNA oligonucleotides for cloning and RT-PCR were purchased from Syntol (Moscow, Russia) and Evrogen (Moscow, Russia).

The list of COL7A1-specific primers is as follows:

Ex3fwGCTGACCATGTCTTCCTGCCC; Ex6revTGTGACTGGCTACAAGGTCCAGT; Ex5-4 rev TCTTGATCCCCACAGCAAATAG; Ex 8-9 fw CGGGTCCTCAGTGGTGGGCCCACACA; Ex 10-9 rev TCAACAGAAGCGTCAGTGCGAGCCATCA; Ex 72 fw GCCCGAACGGCGTCGAGG; Ex 76 rev TGGAGAACAGGGACCCCCTG; Ex 116 rev GTCAGTGCAGCTTCTCCCTT; Ex 108 dir CCCCAGTGGAAATGATGGCT.

Several primers for housekeeping genes and DEGs were used in RT-PCR analysis of expression:

GAPDH fw TGCACCACCAACTGCTTAGC; GAPDH rv GGCATGGACTGTGGTCATGAG; RPL27 fw ACCGCTACCCCCGCAAAGTG; RPL27 rv CCCGTCGGGCCTTGCGTTA; TGM2 fw ATTCCCTCTCCTGCCCAGAT; TGM2 rv AGGGAGCTGGATTCCCTGAT; FN Trf 9fw TCGTGCTTTGACCCCTACAC; FN Trf9 rv CGGGAATCTTCTCTGTCAGCC; CTSZ fw GTCCGGAATTCATGGGGTG; CTSZ rv CTGTGTCGCAGGTCACTTCT; CNN1 fw CAGAGAAGCAGGAGCGGAAA; CNN1 rv CTG GGT ACT CGG GAG TCA G; PPARG fw GACAACCTGCTACAAGCCCT; PPARG rv TTGGCAAACAGCTGTGAGGA; SMAD7 fw CTCTGCGAACTAGAGTCTC; SMAD7 rv ACGCACCAGTGTGACCGA

**Mycoplasma Detection**

Absence of contamination of fibroblasts by mycoplasma was determined by a Myco Real-Time PCR kit (Evrogen, Moscow, Russia) following the manufacturer’s instructions.

**List of Reagents and Equipment for Western Blot Analysis**

Tris(hydroxymethyl)aminomethane (Tris) (VWR Chemicals, Radnor, U.S.), EDTA (Sigma-Aldrich, St. Louis, U.S.), EGTA (Fluka Analytical, ‎Buchs, Switzerland), acrylamide:N,N-methylenebisacrylamide 32.5:1 (Fluka Analytical, ‎Buchs, Switzerland), Glycine (Sigma-Aldrich, St. Louis, U.S.), Tween 20 (Sigma-Aldrich, St. Louis, U.S.), sodium deoxycholate (Sigma-Aldrich, St. Louis, U.S.), sodium dodecyl sulfate (SDS) (Sigma-Aldrich, St. Louis, U.S.), sodium chloride (NaCl) (Sigma-Aldrich, St. Louis, U.S.), PMSF (Sigma-Aldrich, St. Louis, U.S.), 2x Laemmli sample buffer (Bio-Rad, Hercules, U.S.), 2-mercaptoethanol (Sigma-Aldrich, St. Louis, U.S.), urea (Sigma-Aldrich, St. Louis, U.S.), Precision Plus Protein™ Dual Xtra Prestained Protein Standards (Bio-Rad, Hercules, U.S.), Tris–glycine–SDS buffer (Bio-Rad, Hercules, U.S.), ethanol (Konstanta-Farm M, Moscow, Russia ), acetic acid (Dr.Ehrenstorfer, Augsburg, Germany), Hybond^®^ ECL^™^ nitrocellulose membranes (Sigma-Aldrich, St. Louis, U.S.), Immun-Blot^®^ LF polyvinylidene difluoride (PVDF) 0.45 μm membrane (Bio-Rad, Hercules, U.S.), blotting-grade blocker nonfat dried milk powder (Bio-Rad, Hercules, U.S.), goat polyclonal anti-collagen VIIa antibodies (Thermofisher, Waltham, U.S., PA5-18390), rabbit polyclonal anti-β-actin antibodies (Abcam, Cambridge, U.K., ab1801), rabbit monoclonal anti-hTERT antibodies (Abcam, Cambridge, U.K. ab32020), HRP-conjugated anti-rabbit antibodies (Bio-Rad, Hercules, U.S., 170-6515), polyclonal goat antibodies to NC1 type VII collagen peptide (Genetex, Irvine, U.S., GTX89040), ImmPRESS® HRP Horse Anti-Goat IgG Polymer Detection Kit, peroxidase (Vector Laboratories, Burlingame, U.S., MP-7405), Clarity Western ECL Substrate (Bio-Rad, Hercules, U.S.), Trans-blot Turbo Transfer system (Bio-Rad, Hercules, U.S.), ChemiDocMP Imaging System (Bio-Rad, Hercules, U.S.), NanoPhotometer P360 (Implen, Munich, Germany).

**WB of Type VII Collagen**

Fibroblasts were harvested using 0.05% trypsin–EDTA solution (Gibco, Grand Island, New York, USA), transferred to a 1.5 mL PCR tube, pelleted by centrifugation, washed with PBS (PanEco, Moscow, Russia) and lysed in RIPA buffer (50 mM Tris, 10 mM EDTA, 0.5 mM EGTA, 1% Tween 20, 0.1% sodium deoxycholate, 0.1% SDS, 150 mM NaCl, 8 M urea, 1 mM PMSF added before use). The lysates were incubated on ice for 1 h with occasional vortexing, then centrifuged at 12000 rcf for 10 min at 4 °C. The supernatants were transferred to new tubes. Protein concentration was determined by measuring absorbance at 280 nm. Lysates were diluted 1:1 with 2x Laemmli sample buffer (2-mercaptoethanol added before use) and incubated for 5 min at 95 °C.

Samples (70 μg protein) were loaded and separated on denaturing 8% SDS–Tris–glycine polyacrylamide gel (PAAG) with 8 M urea along with a molecular weight marker (161-0377, Bio-Rad, Hercules, California, USA) or DsRed fluorescent protein expressed in the pQE30 plasmid as described in [1]. The part of the gel with bands approximately higher than 100 kDa was washed at room temperature according to the protocol: 1% acetic acid water solution with 10% EtOH for 20 min, 2% acetic acid water solution with 5% EtOH for 15 min, 2% acetic acid water solution for 15 min, 3% acetic acid water solution for 15 min 2 times. Then, proteins were semidry-electrotransferred (20 V, 120 min) onto nitrocellulose membrane (the gel was under the membrane in the transfer sandwich) at 4 °C in cold 3% acetic acid water solution, then washed with Tris-buffered saline with 0.1% Tween-20 (TBST) 3 times for 5 min, blocked with 3% nonfat dried milk powder in TBST (blocking buffer) for 1 h, washed 3 times for 10 min with TBST and incubated with primary anti-collagen VII antibodies (PA5-18390, Invitrogen, Carlsbad, California, USA) dissolved in blocking buffer overnight at 4 °C. Horseradish peroxidase (HRP)-conjugated anti-goat secondary antibodies (MP-7405, Vector Laboratories, Burlingame, California, USA) solution was used for immunodetection. Blots were imaged by ECL and the signal was detected using a ChemiDoc MP Imaging System. Data was generated with the software Image Lab 5.0 (Bio-Rad, Hercules, California, USA) and evaluated using the Fiji software function ‘Analyse > Gels’ [2]. This function was also used to detect low-intensity bands by using it on a single lane. Protein weight estimation was made using a linear plot of markers’ molecular weight in logarithmic scale and the relative migration distance of the target protein [3].

1[. Verkhusha, V.V.; Chudakov, D.M.; Gurskaya, N.G.; Lukyanov, S.; Lukyanov, K.A. Common Pathway for the Red Chromophore Formation in Fluorescent Proteins and Chromoproteins. *Chem. Biol.* **2004**, *11*, 845–854, doi:10.1016/j.chembiol.2004.04.007.](https://www.zotero.org/google-docs/?t7TI80)

2[. Gels [ImageJ Documentation Wiki] Available online: https://imagejdocu.tudor.lu/gui/analyze/gels (accessed on 29 December 2020).](https://www.zotero.org/google-docs/?t7TI80)

3[. Man, T.P. Determining Protein Molecular Weight with SDS-PAGE: An Overview of the Process Available online: https://info.gbiosciences.com/blog/bid/196974/determining-protein-molecular-weight-with-sds-page-an-overview-of-the-process (accessed on 29 December 2020).](https://www.zotero.org/google-docs/?t7TI80)
