# Supplementary material for: Signatures of Dermal Fibroblasts from RDEB Pediatric Patients
Source: Int J Mol Sci. 2021 Feb 11;22(4):1792. doi: 10.3390/ijms22041792 (PMC7918539; doi:10.3390/ijms22041792)
Supplement: Supplementary file 1 [file ijms-22-01792-s001.zip › ijms-1080037-proofed-supplementary/Supplementary Results-edited.docx]

**Supplementary Results**


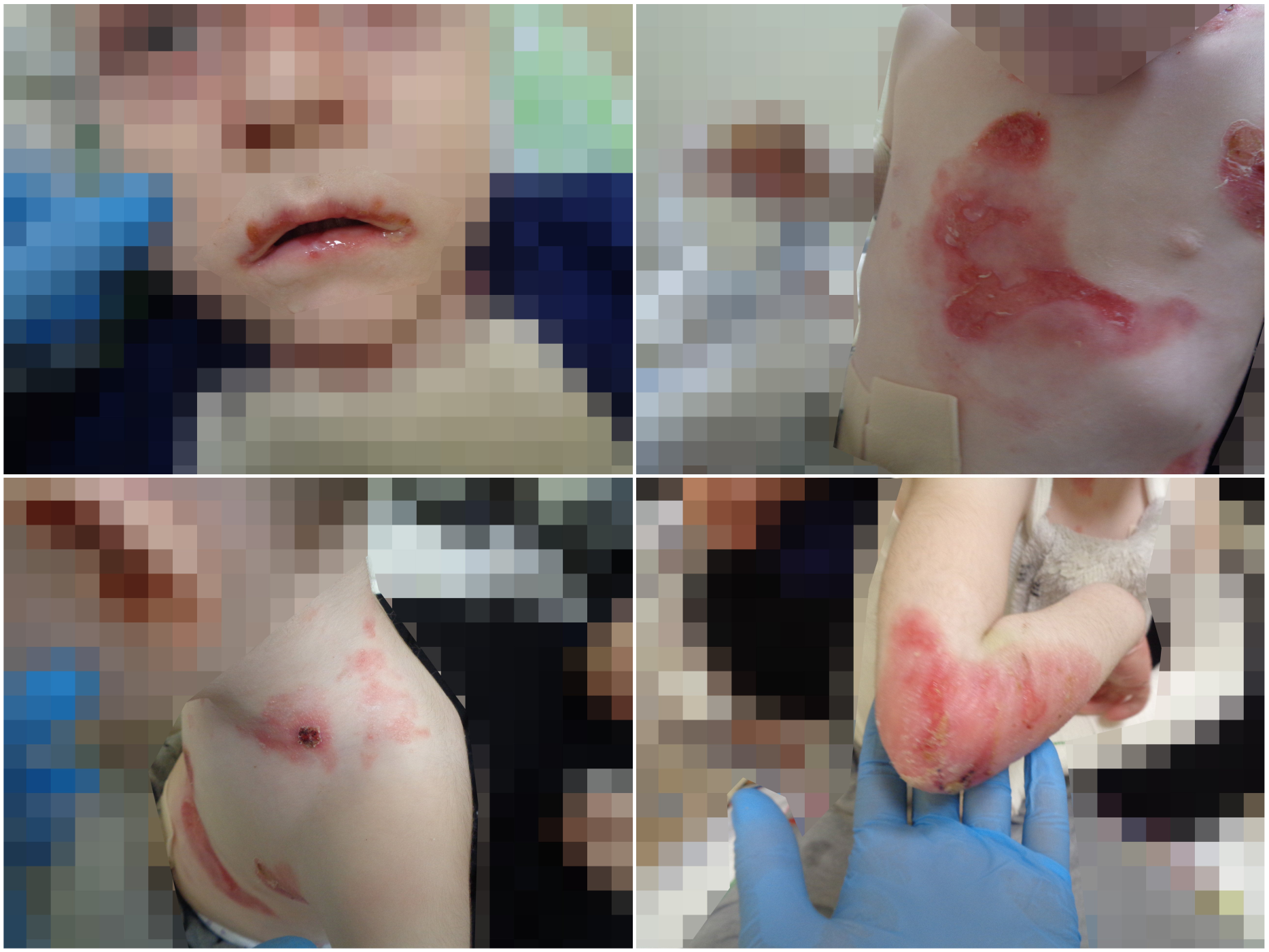


**Figure S1.** Manifestation of skin lesions in patient d3 with a generalized severe form of RDEB. The skin pathological process was widespread, represented by spots, blisters, milia, erosion, scales, crusts and scars, localized on the skin of the scalp, face, neck, trunk, upper and lower limbs. The patient subjectively experienced itching and soreness.


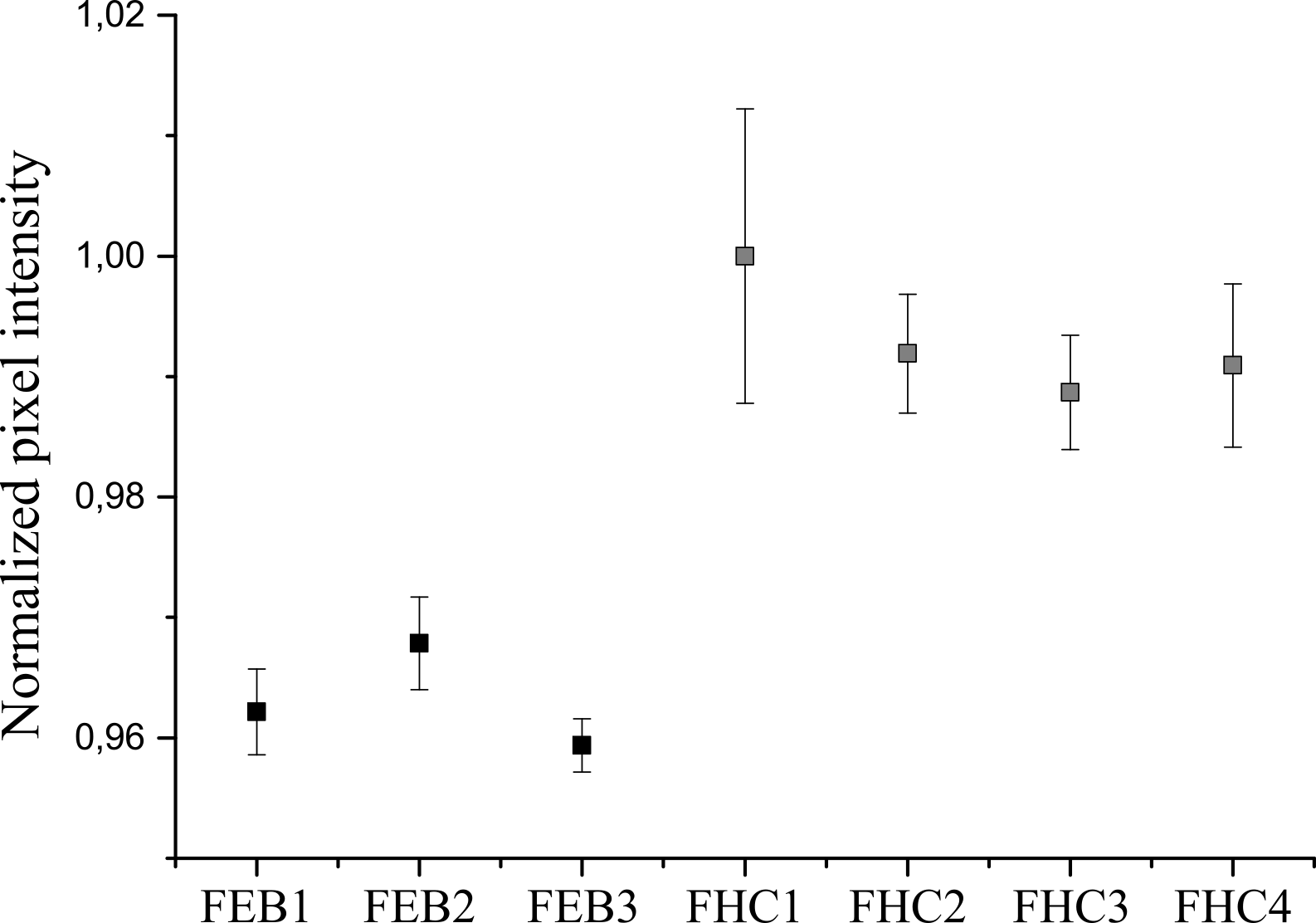


**Figure S2**. Type VII collagen expression level in FEB and FHC lines. Mean values and standard error are shown for each cell line. The highest mean value was selected for each. A detailed description of the processing of primary data is presented in Section 4.11.

**Table S1.** The results of the nested t test comparing type VII collagen expression of RDEB and normal fibroblast cells.

| **P value** | **<0.0001** |
| --- | --- |
| Significantly different (P < 0.05)? | Yes |
| One- or two-tailed P value? | Two-tailed |
| Mean of EB-FB | 0.9837 |
| Mean of N-FB | 1.014 |
| Difference between means: (A − B) ± SEM | -0.03038 ± 0.004674 |
| 95% confidence interval | −0.03999 to −0.02078 |

**2S.1.** **Splice Site Prediction in FEB Lines**

The transition *c.425A>G* (p.K142R) placed “G” in the “−2” position of the exon 3 splicing site. Indeed, this position of donor splice sites was occupied by a highly conserved “A” in almost all metazoan genes [1]. The pathology revealed in the case of this mutation was determined by the expression of three alternative transcripts carrying a PTC [2]. We checked the splicing efficiency in silico with the help of the splice site prediction resource on the BDGP Information platform [3]. The tool confirmed the absence of a donor splicing site of exon 3 *COL7A1* gene that should impair the correct splicing (Table 2S).

The first mutation of patient d2, *c.682+1G>A*, was located in intron 5, as described first in [4], where it presumably disrupted the donor splice site. With the help of NNSPLICE, we checked the splicing efficiency of the *COL7A1* mutant in patient d2 (Table 2S).

**Table S2.** Splice site prediction by NNSPLICE on *COL7A1* *c.425A>G*, *c.682+1G>A* and *c.520G>A* templates in comparison with wild-type *COL7A1*.

| **Start** | **End** | **Exon Intron *COL7A1* *Gene ID*: *1294*** | **Score** | **Start** | **End** | **Exon Intron *COL7A1***  ***c.425A>G*** | **Score** |
| --- | --- | --- | --- | --- | --- | --- | --- |
| 1241 | 1255 | ccttcagGTctgccc | 0.43 | 1241 | 1255 | cttcagGTctgccc | 0.43 |
| 1617 | 1631 | ccccaagGTgatccc | 0.84 | 1617 | 1631 | - | - |
| 1798 | 1812 | gctgtggGTaaggac | 0.92 | 1798 | 1812 | gctgtggGTaaggac | 0.92 |
| 1822 | 1836 | gtgacagGTcagctg | 0.68 | 1822 | 1836 | gtgacagGTcagctg | 0.68 |
| **Start** | **End** | **Exon Intron *COL7A1* *Gene ID*: *1294*** | **Score** | **Start** | **End** | **Exon Intron *COL7A1* *c.682+1G>A*** | **Score** |
| 2327 | 2341 | ccccaagGTgatccc | 0.84 | 1949 | 1963 | cttcagGTctgccc | 0.43 |
| 2504 | 2518 | atttgctGTgagtaa | 0.57 | 2504 | 2518 | atttgctGTgagtaa | 0.57 |
| 1822 | 1836 | gtgacagGTcagctg | 0.68 | 1822 | 1836 | gtgacagGTcagctg | 0.92 |
| 2052 | 2066 | cgacctcGTgagttc | 0.90 | 2052 | 2066 | - | - |
| 2379 | 2393 | gcaggagGTaggatg | 0.78 | 2379 | 2393 | gcaggagGTaggatg | 0.68 |
| **Start** | **End** | **Exon Intron *COL7A1* *Gene ID*: *1294*** | **Score** | **Start** | **End** | **Exon Intron *COL7A1* *c.520G>A*** | **Score** |
| 1617 | 1631 | ccccaagGTgatccc | 0.84 | 1617 | 1631 | ccccaagGTgatccc | 0.84 |
| 1798 | 1812 | gctgtggGTaaggacgg | 0.92 | 1794 | 1808 | atttgctGTgagtaaggatca | 0.57 |
| 1822 | 1836 | gtgacagGTcagctg | 0.68 | 1822 | 1836 | gtgacagGTcagctg | 0.68 |
| 2052 | 2066 | cgacctcGTgagttc | 0.90 | 2052 | 2066 | cgacctcGTgagttc | 0.90 |

*Start and End columns indicate the COL7A1 base pairs from the start codon of COL7A1 (the 171th nucleotide of the NC_000003.12NCBI reference sequence). Upper register of base pairs (GT) indicates the beginning of the intron. Score represents the relativeness of the splicing site.

The NNSPLICE program predicts the absence of a donor splice site for the *c.682+G>A* mutation but leaves the acceptor site exactly the same as in the wild-type exon 6 of *COL7A1* (Supplementary Table S2). Due to the abnormal splicing, the open reading frame leads to a PTC 128 nucleotides upstream of exon 6. The NMD in this case presumably results in the protein deficiency. The second mutation of the subject is *c.6205C>T* of exon 74, corresponding to the p.R2069C missense mutation in the collagenous domain of COL7A1 (Figure S2, B). This mutation was described as recessive and is responsible for acquiring the phenotype of inversa-type RDEB [5–7].

The fourth patient (d4) had the pathological *c.520G>A* transition, which is the known glycine missense mutation that may cause RDEB as well as the DDEB type of EB [8]. This transition destroys the normal donor splice site according to the NNSPLICE prediction (Supplementary Table S2). The alternative cryptic donor site is located upstream, carries the PTC and has a low score, according to the prediction.

**2S.2.** **Mutations in Patient D3**

The FEB3 line had a rare combination of two missense mutations: a homozygous mutation in *COL7A1* and a heterozygous mutation in KRT5. In this case, RDEB is caused by the missense mutation *c.8245G>A* that leads to the glycine substitution p.G2749R (Supplementary Figure S4, D). The new heterozygous mutation in *KRT5* was identified in our study, c.1054C>T (OMIM 148040), corresponds to the substitution p.R352C (Supplementary Figure 4S, E). This Arg to Cys substitution has not been detected before, although the Arg to Ser substitution R352S in *KRT5* has been described previously [9]**.**


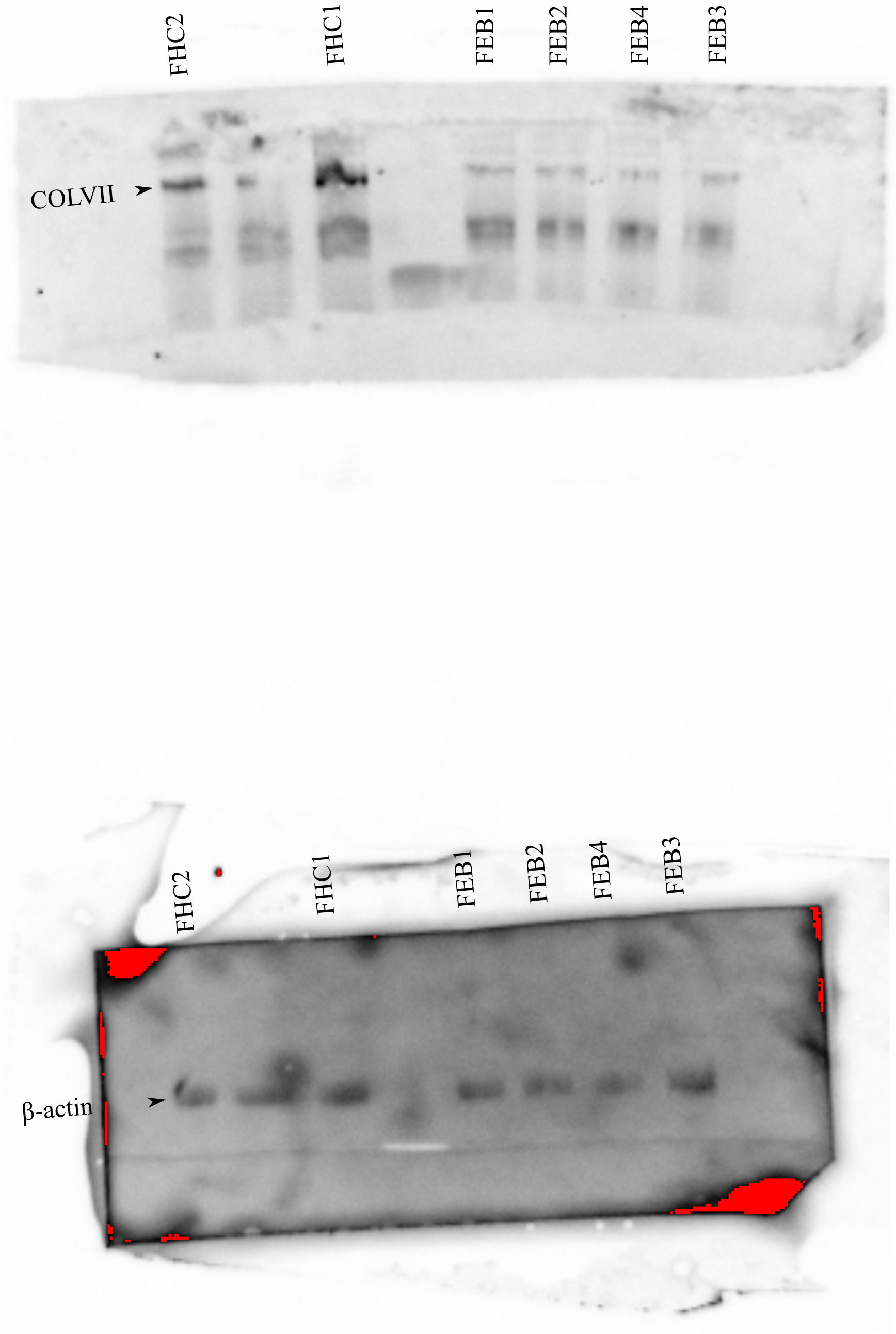


**Figure S3.** Uncut and unadjusted Western blot of total cell lysates. SDS-PAGE (8%) with 8 M urea, anti-type-VII collagen polyclonal antibody (upper panel), anti-β-actin (lower panel), ECL detection**.**


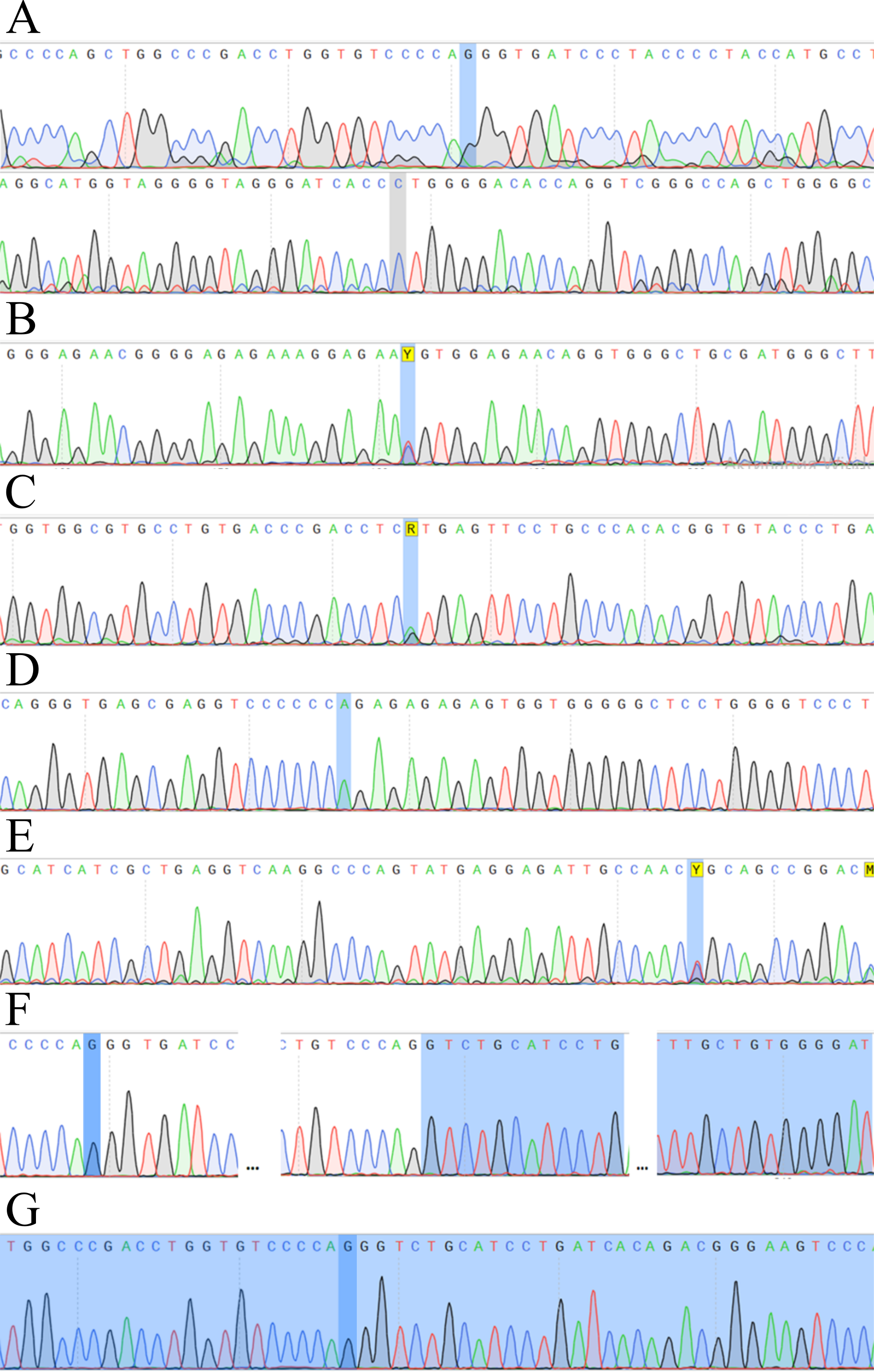


**Figure S4.** Local context of mutations uncovered. A. d1FEB1 COL7A1 c.425A>G. Homozygous missense mutation p. K142R (forward and reverse chains) B. d2 FEB2 COL7A1 c.6205C>T. Heterozygous missense mutation p. R2069C C. d2FEB2 COL7A1 c.682+1G>A. Heterozygous mutation in intron 5 D. d3FEB3 COL7A1c.8245G>A. Homozygous mutation p.G2749R E. d3FEB3 KRT5 c.1054C>T. Heterozygous mutation p.R352C F. Sequence of COL7A1 FEB1cDNA, fragment COL7A1 (form with retained intron). Three fragments of the sequence are represented: 1) the exon 3 with c.425A>G, which is indicated by dark blue color, followed by the intron sequence; 2) the retained intron and the beginning of exon 4; 3) the junction site of exon 4 and exon 5. Darker blue color indicates the mutation site c.425A>G and the exonic sequences on the second and third fragments of the sequence. G. Sequence of FEB1 cDNA: fragment of COL7A1 (c.425A>G) with normal splicing. Darker blue color indicates the site c.425A>G, which is fused with the following sequence of exon 4. The open reading frame of COL7A1 is highlighted and not interrupted.


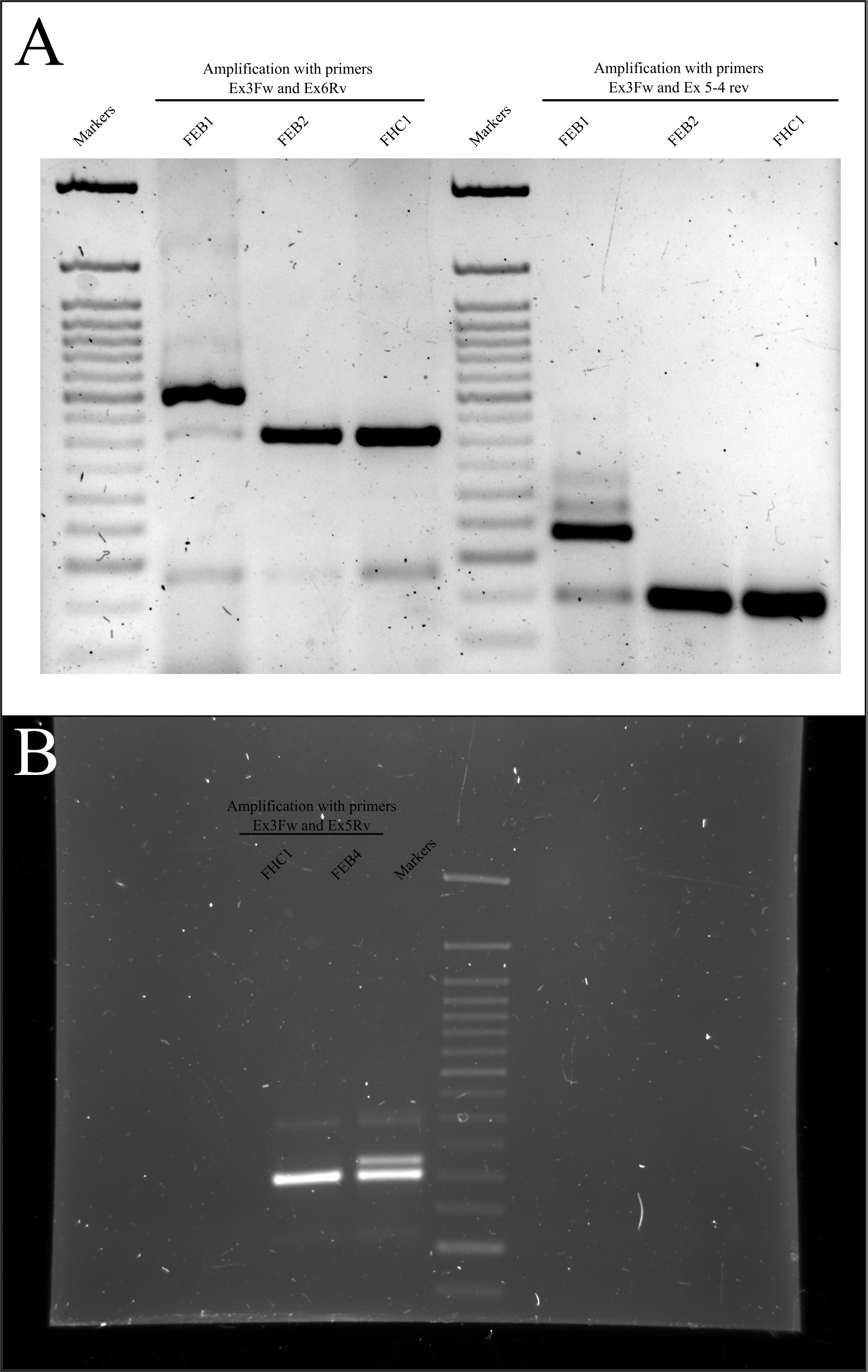


**Figure S5.** Uncut and unadjusted images of RT-PCR analysis of COL7A1.

**2S.3.** **Mutations Influence COL7A1 mRNA Processing**

The mutation *c.425A>G* (genome position 48650971 in GRCh37/hg19) was found to be recurrent for the RDEB European population. It was first described as an example of a splice mutation in DEB [2], and further pathogenicity of the donor splice site dropping-out was confirmed for the heterozygous state of this mutation [5–7]. Patient d1 had the mutation *c.425A>G* in the homozygous state, giving us the opportunity to study the work of the splicing machinery for this mutation. Despite the predicted absence of the donor splicing site for intron 3, the normal splice product was shown to be present, though sparsely, in FEB1 cells (Figure 4A). The sequencing of the bands confirmed the identity of two spliced forms of the transcript: the original spliced variant of the transcript (Supplementary Figure S4, E) and the transcript with retained intron 3 (Supplementary Figure S4, D). We showed that normal splicing events take place in the cells despite the donor site destruction in FEB1, and this finding could be important for understanding how the local sequence context near the mutation may influence the splicing process. The product of normal splicing with *c.425A>G* carries the amino acid substitution Lys to Arg, which appears to be a conservative mutation. The translation of this transcript could result in a protein without significant disturbances of the spatial structure [10]. Therefore, the trace amounts of full-length C7 protein are capable of being produced in FEB1. The second aberrant form with retained intron 3 contained a PTC 18 nt upstream of the exon junction (EJ) site, making the PTC unrecognizable by NMD machinery, according to the “50–55 nt rule” [11,12].

As noted before, the “leaking'' abnormal splice site with *c.425A>G* may be linked to the synthesis of correctly spliced *COL7A1* mRNA in minor amounts. The appearance of a PTC not associated with RNA degradation has been shown earlier for dominant negative *COL7A1* mutations [13], where the PTC appeared in *COL7A1* after targeting by the programmed nuclease. We hypothesize that this type of PTC could potentially be used in the development of the gene therapy of DEB. All things considered, in order to better understand the pathogenicity of the mutation, it is important to evaluate the position of the PTC in the local exonic–intronic context.

The *c.425A>G* mutation attracts attention not only because of its recurrent frequency in the European population, but also because of its potential of being cured by the CRISPR/Cas9 gene editing system. The local sequence context around the site of the mutation contains a protospacer adjacent motif (PAM) sequence for active single guide RNA targeting and has been successfully applied in gene therapy [10,14].

In contrast to the *с.425A>G* mutation in patient d1, the NMD mechanism was active in FEB2 for the first mutation of *COL7A1* *c.682+1G>A* (genome position 48630534 in GRCh37/hg19). This mutation disrupts the donor splice site and was described previously [15]**.** The recognition of the PTC located in the retained intron 6 at 100 nt upstream of EJ leads to the successful degradation of this mutant transcript. However, the predicted splicing impairment in the FEB2 line (Supplementary Table S2) was not detected by RT-PCR nor by the sequencing of cDNA fragment mixtures. We suggest that the NMD decay prevented us from obtaining the fragments of such a transcript. Indeed, we have only observed normal splicing events in FEB2 cells, which take place after the transcription from the other allele.

The *c.520G>A COL7A1* mutation in patient d4 (genome position 48630789 in GRCh37/hg19) was described earlier [8] in a patient with Hallopeau-Siemens typeRDEB, where this mutation was combined with the heterozygous mutation in exon 117. The initial description of *c.520G>A* as simply the missense mutation p.G174R was revised later, after the significance of pathological splicing impairment was shown. According to the in silico splice site prediction, we report the splicing disturbance for *c.520G>A* due to the absence of a donor splicing site of exon 4, namely the disrupted “−2” position of the donor splice site (Table S2). Cryptic donor site activation was also predicted with a relatively low score (0.57). The splicing event results in the appearance of the transcript with a PTC susceptible to NMD degradation. RT-PCR analysis revealed the form with the retained intron as well as the original shorter form (Figure 4B, the longer band corresponds to this mutant transcript).

Thus, PTC is present in FEB2 and FEB4 transcripts, in both cases activating the NMD mechanism, which recognizes and destroys the major forms of the transcripts. NMD was shown to be dependent on sequence context and cell type. Clearly, it is important to uncover whether the position of the PTC correlates with the activity of NMD as well as with the clinical severity of RDEB [12,16].

FEB3 cells have the homozygous mutation p.G2749R of COL7A1 that causes a severe form of RDEB combined with the new mutation in KRT5, p.R352C. No splicing impairments were detected in this case. The impact of p.G2749R on the disturbance of C7 structure was shown previously by the exogenous expression of the recombinant protein [17]. This mutant form of C7 has a decreased ability to fold into its trimer form and has increased sensitivity to protease degradation.


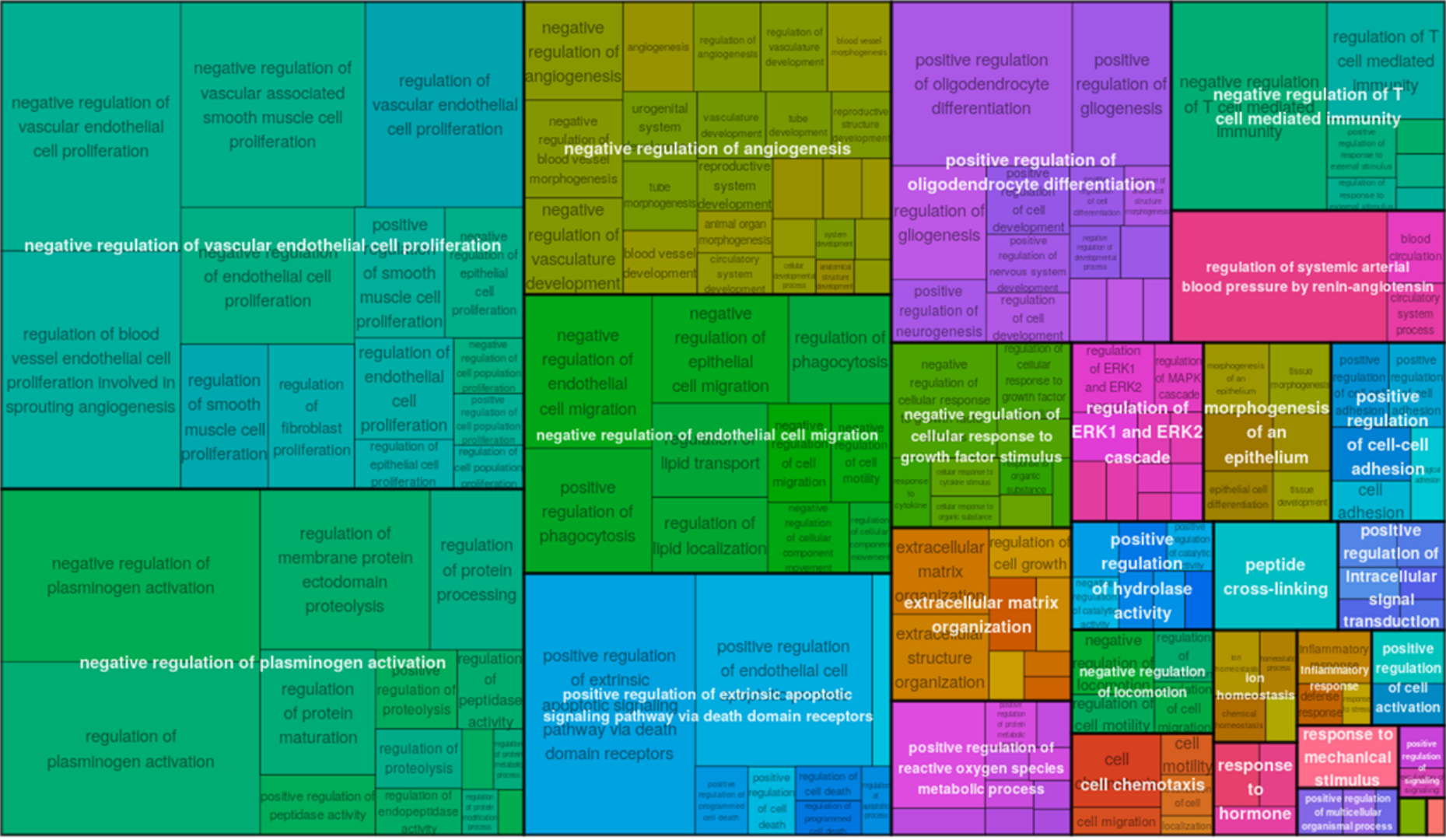


**Figure S6 A.** Tree map of gene ontology (GO) biological processes (BP) terms of DEGs found in RT-qPCR data of FEB and FHC lines. The area of each square is proportional to the fold enrichment of the respective terms.


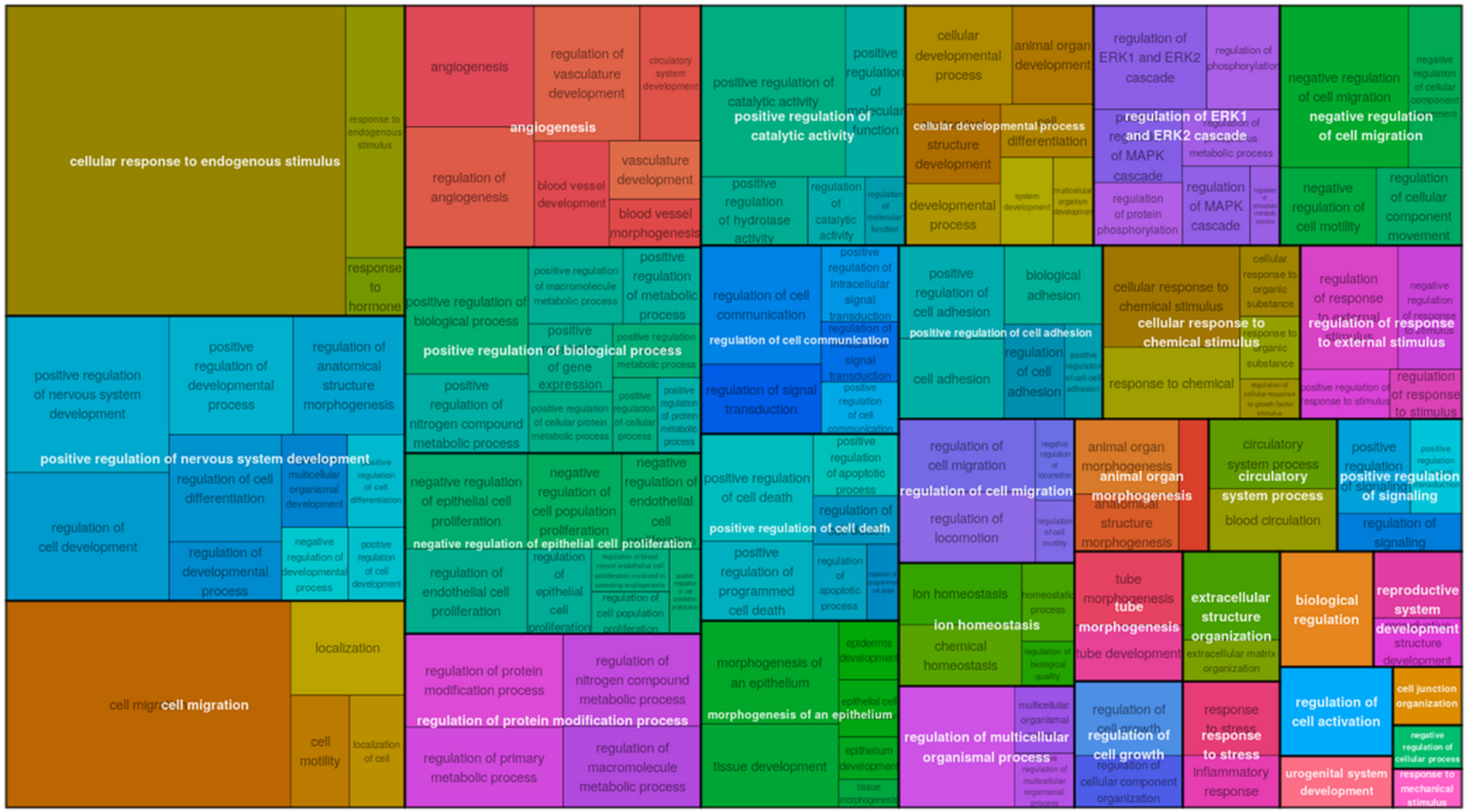


**Figure S6 B.** Tree map of common GO terms of DEGs found in RDEB RNA-seq and RT-qPCR data of FEB and FHC lines. The area of each square is proportional to the fold enrichment of the respective terms.


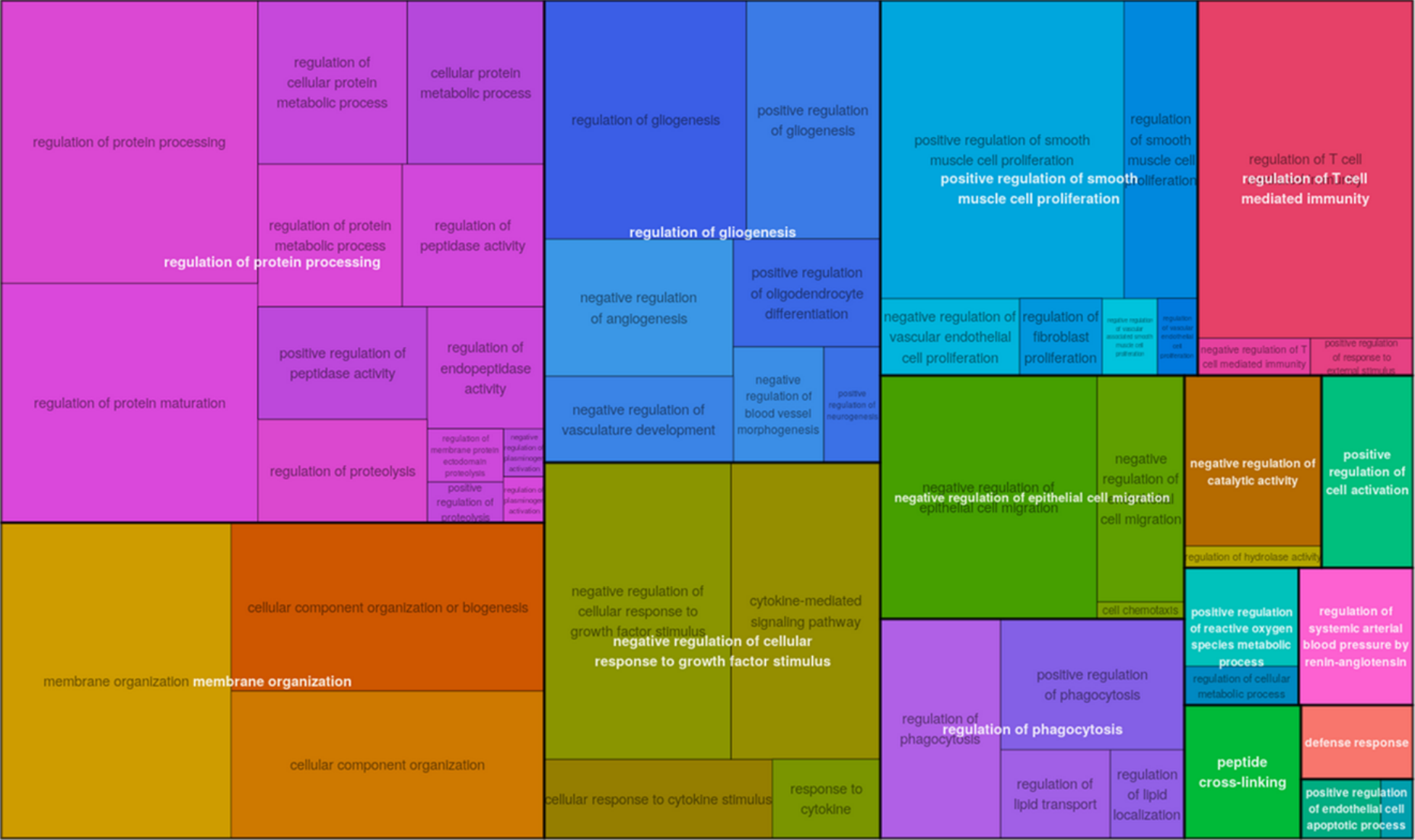


**Figure S6 C.** Tree map of the unique GO terms of DEGs found in RT-qPCR data of FEB and FHC lines. The area of each square is proportional to the fold enrichment of the respective terms.


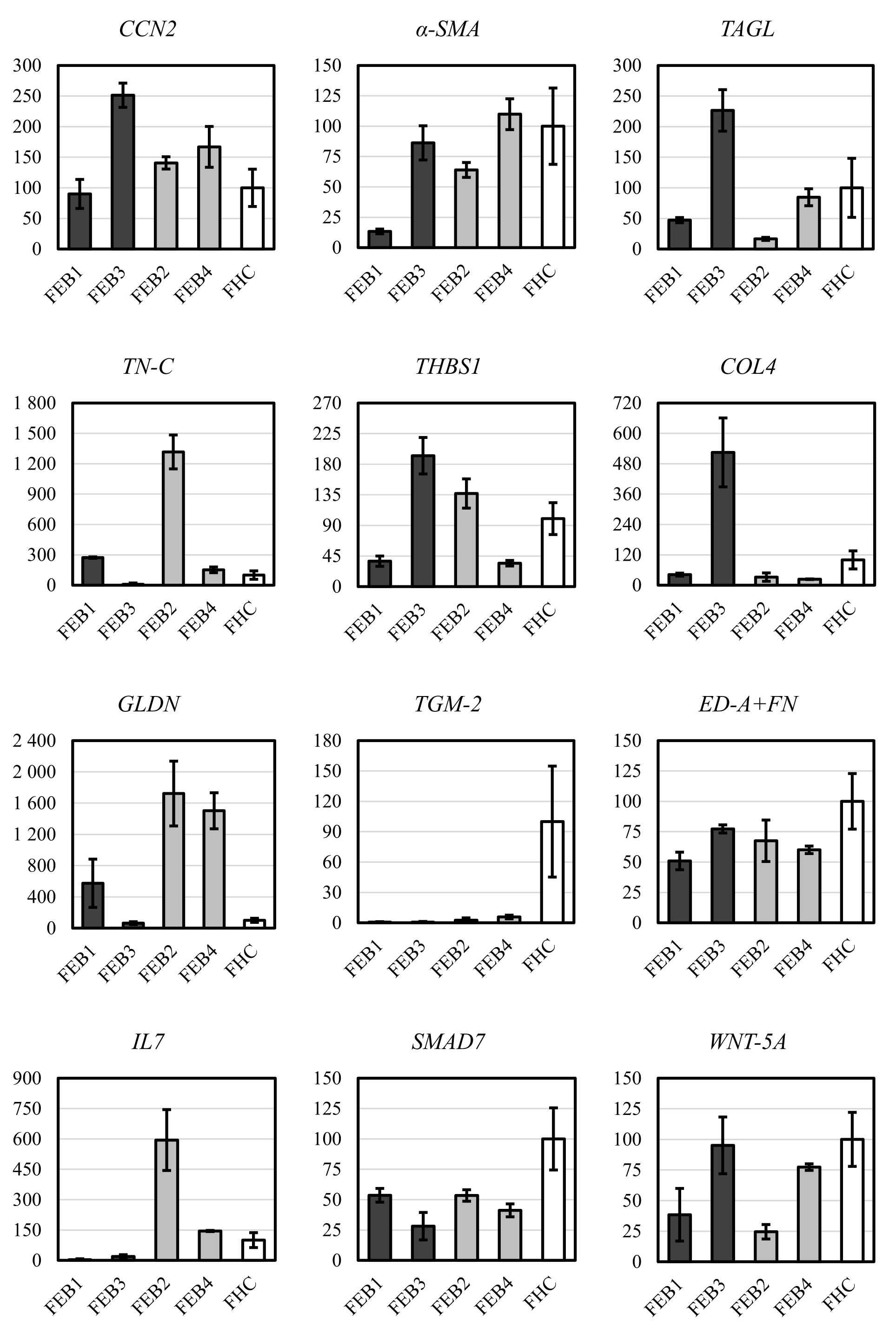


**Figure S7.** RT-qPCR analysis of DEGs in FEB and FHC lines. Dark grey and light grey column colors indicate cell lines from patients with severe (FEB1, FEB3) and mild (FEB2, FEB4) forms of EB, respectively. White column color indicates the healthy control. The ordinate value is U—relative gene expression, estimated as ΔΔCT. The mean value is given for each FEB line and the average value of pooled data from several FHC lines. Error is the 95% confidence interval (CI).

[1. Denisov, S.; Bazykin, G.; Favorov, A.; Mironov, A.; Gelfand, M. Correlated Evolution of Nucleotide Positions within Splice Sites in Mammals. *PLOS ONE* **2015**, *10*, e0144388, doi:10.1371/journal.pone.0144388.](https://www.zotero.org/google-docs/?t7TI80) [82. BDGP: Splice Site Prediction by Neural Network Available online: https://www.fruitfly.org/seq_tools/splice.html (accessed on 29 December 2020).](https://www.zotero.org/google-docs/?t7TI80)

[2. Gardella, R.; Belletti, L.; Zoppi, N.; Marini, D.; Barlati, S.; Colombi, M. Identification of Two Splicing Mutations in the Collagen Type VII Gene (COL7A1) of a Patient Affected by the Localisata Variant of Recessive Dystrophic Epidermolysis Bullosa. *Am. J. Hum. Genet.* **1996**, *59*, 292–300.](https://www.zotero.org/google-docs/?t7TI80)

3[. BDGP: Splice Site Prediction by Neural Network Available online: https://www.fruitfly.org/seq_tools/splice.html (accessed on 29 December 2020).](https://www.zotero.org/google-docs/?t7TI80)

4[. Hovnanian, A.; Rochat, A.; Bodemer, C.; Petit, E.; Rivers, C.A.; Prost, C.; Fraitag, S.; Christiano, A.M.; Uitto, J.; Lathrop, M.; et al. Characterization of 18 New Mutations in COL7A1 in Recessive Dystrophic Epidermolysis Bullosa Provides Evidence for Distinct Molecular Mechanisms Underlying Defective Anchoring Fibril Formation. *Am. J. Hum. Genet.* **1997**, *61*, 599–610, doi:10.1086/515495.](https://www.zotero.org/google-docs/?t7TI80)

5[. Hamidi, A.K.; Moghaddam, M.; Hatamnejadian, N.; Ebrahimi, A. A Novel Deletion and Two Recurrent Substitutions on Type VII Collagen Gene in Seven Iranian Patients with Epidermolysis Bullosa. *Iran. J. Basic Med. Sci.* **2016**, *19*, 858–862.](https://www.zotero.org/google-docs/?t7TI80)

6[. Kocher, T.; Wagner, R.N.; Klausegger, A.; Guttmann-Gruber, C.; Hainzl, S.; Bauer, J.W.; Reichelt, J.; Koller, U. Improved Double-Nicking Strategies for COL7A1-Editing by Homologous Recombination. *Mol. Ther. - Nucleic Acids* **2019**, *18*, 496–507, doi:10.1016/j.omtn.2019.09.011.](https://www.zotero.org/google-docs/?t7TI80)

7[. Dang, N.; Klingberg, S.; Marr, P.; Murrell, D.F. Review of Collagen VII Sequence Variants Found in Australasian Patients with Dystrophic Epidermolysis Bullosa Reveals Nine Novel COL7A1 Variants. *J. Dermatol. Sci.* **2007**, *46*, 169–178, doi:10.1016/j.jdermsci.2007.02.006.](https://www.zotero.org/google-docs/?t7TI80)

[8. Whittock, N.V.; Ashton, G.H.; Mohammedi, R.; Mellerio, J.E.; Mathew, C.G.; Abbs, S.J.; Eady, R.A.; McGrath, J.A. Comparative Mutation Detection Screening of the Type VII Collagen Gene (COL7A1) Using the Protein Truncation Test, Fluorescent Chemical Cleavage of Mismatch, and Conformation Sensitive Gel Electrophoresis. *J. Invest. Dermatol.* **1999**, *113*, 673–686, doi:10.1046/j.1523-1747.1999.00732.x.](https://www.zotero.org/google-docs/?t7TI80)

9[. Yasukawa, K.; Sawamura, D.; Goto, M.; Nakamura, H.; Jung, S.-Y.; Kim, S.-C.; Shimizu, H. Epidermolysis Bullosa Simplex in Japanese and Korean Patients: Genetic Studies in 19 Cases. *Br. J. Dermatol.* **2006**, *155*, 313–317, doi:10.1111/j.1365-2133.2006.07285.x.](https://www.zotero.org/google-docs/?t7TI80)

10[. Zhang, Z.; Miteva, M.A.; Wang, L.; Alexov, E. Analyzing Effects of Naturally Occurring Missense Mutations. *Comput. Math. Methods Med.* **2012**, *2012*, doi:10.1155/2012/805827.](https://www.zotero.org/google-docs/?t7TI80)

11[. Hentze, M.W.; Kulozik, A.E. A Perfect Message: RNA Surveillance and Nonsense-Mediated Decay. *Cell* **1999**, *96*, 307–310, doi:10.1016/s0092-8674(00)80542-5.](https://www.zotero.org/google-docs/?t7TI80)

12[. Ishiko, A.; Masunaga, T.; Ota, T.; Nishikawa, T. Does the Position of the Premature Termination Codon in COL7A1 Correlate with the Clinical Severity in Recessive Dystrophic Epidermolysis Bullosa? *Exp. Dermatol.* **2004**, *13*, 229–233, doi:10.1111/j.0906-6705.2004.00167.x.](https://www.zotero.org/google-docs/?t7TI80)

1[3. Shinkuma, S.; Guo, Z.; Christiano, A.M. Site-Specific Genome Editing for Correction of Induced Pluripotent Stem Cells Derived from Dominant Dystrophic Epidermolysis Bullosa. *Proc. Natl. Acad. Sci. U. S. A.* **2016**, *113*, 5676–5681, doi:10.1073/pnas.1512028113.](https://www.zotero.org/google-docs/?t7TI80)

14[. March, O.P.; Kocher, T.; Koller, U. Context-Dependent Strategies for Enhanced Genome Editing of Genodermatoses. *Cells* **2020**, *9*, doi:10.3390/cells9010112.](https://www.zotero.org/google-docs/?t7TI80)

15[. Varki, R.; Sadowski, S.; Uitto, J.; Pfendner, E. Epidermolysis Bullosa. II. Type VII Collagen Mutations and Phenotype-Genotype Correlations in the Dystrophic Subtypes. *J. Med. Genet.* **2007**, *44*, 181–192, doi:10.1136/jmg.2006.045302.](https://www.zotero.org/google-docs/?t7TI80)

16[. Linde, L.; Boelz, S.; Nissim-Rafinia, M.; Oren, Y.S.; Wilschanski, M.; Yaacov, Y.; Virgilis, D.; Neu-Yilik, G.; Kulozik, A.E.; Kerem, E.; et al. Nonsense-Mediated MRNA Decay Affects Nonsense Transcript Levels and Governs Response of Cystic Fibrosis Patients to Gentamicin. *J. Clin. Invest.* **2007**, *117*, 683–692, doi:10.1172/JCI28523.](https://www.zotero.org/google-docs/?t7TI80)

17[. Chen, M.; Costa, F.K.; Lindvay, C.R.; Han, Y.-P.; Woodley, D.T. The Recombinant Expression of Full-Length Type VII Collagen and Characterization of Molecular Mechanisms Underlying Dystrophic Epidermolysis Bullosa. *J. Biol. Chem.* **2002**, *277*, 2118–2124, doi:10.1074/jbc.M108779200.](https://www.zotero.org/google-docs/?t7TI80)
