# Supplementary figures and images for: Signatures of Dermal Fibroblasts from RDEB Pediatric Patients
Source: Int J Mol Sci. 2021 Feb 11;22(4):1792. doi: 10.3390/ijms22041792 (PMC7918539; doi:10.3390/ijms22041792)

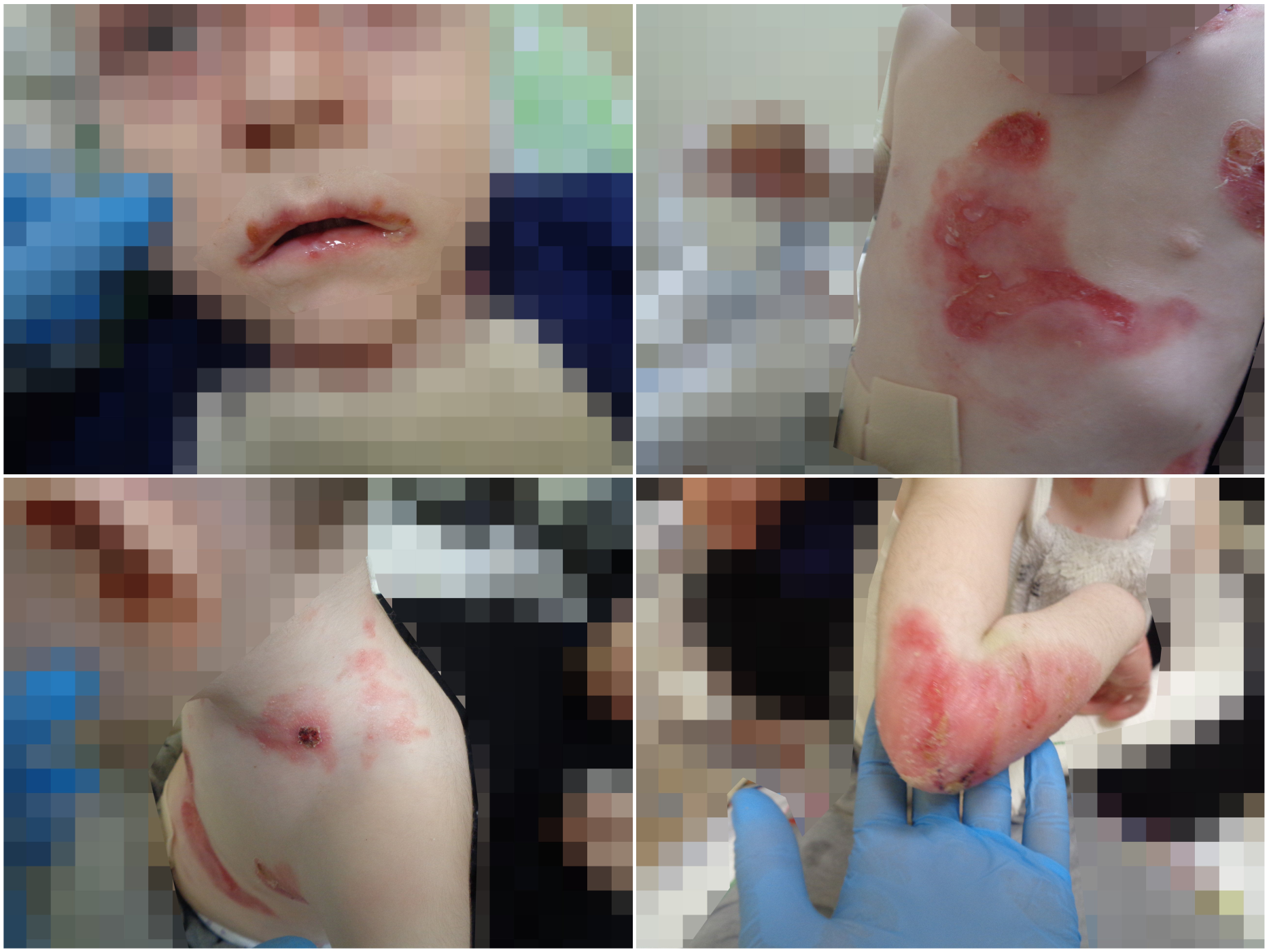

Supplement: Supplementary file 1 [file ijms-22-01792-s001.zip › ijms-1080037-proofed-supplementary/Figure S1.png]

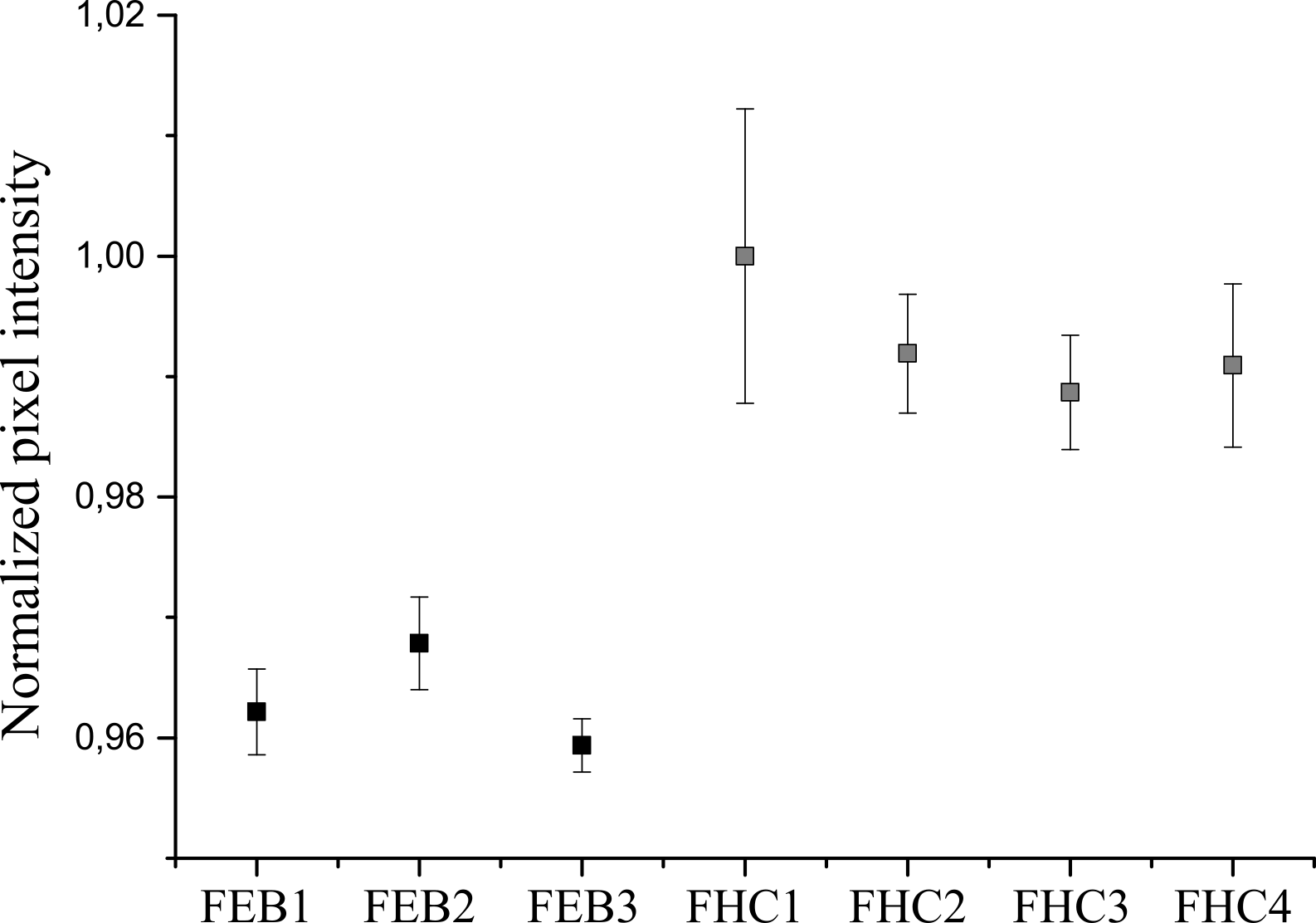

Supplement: Supplementary file 1 [file ijms-22-01792-s001.zip › ijms-1080037-proofed-supplementary/Figure S2.png]

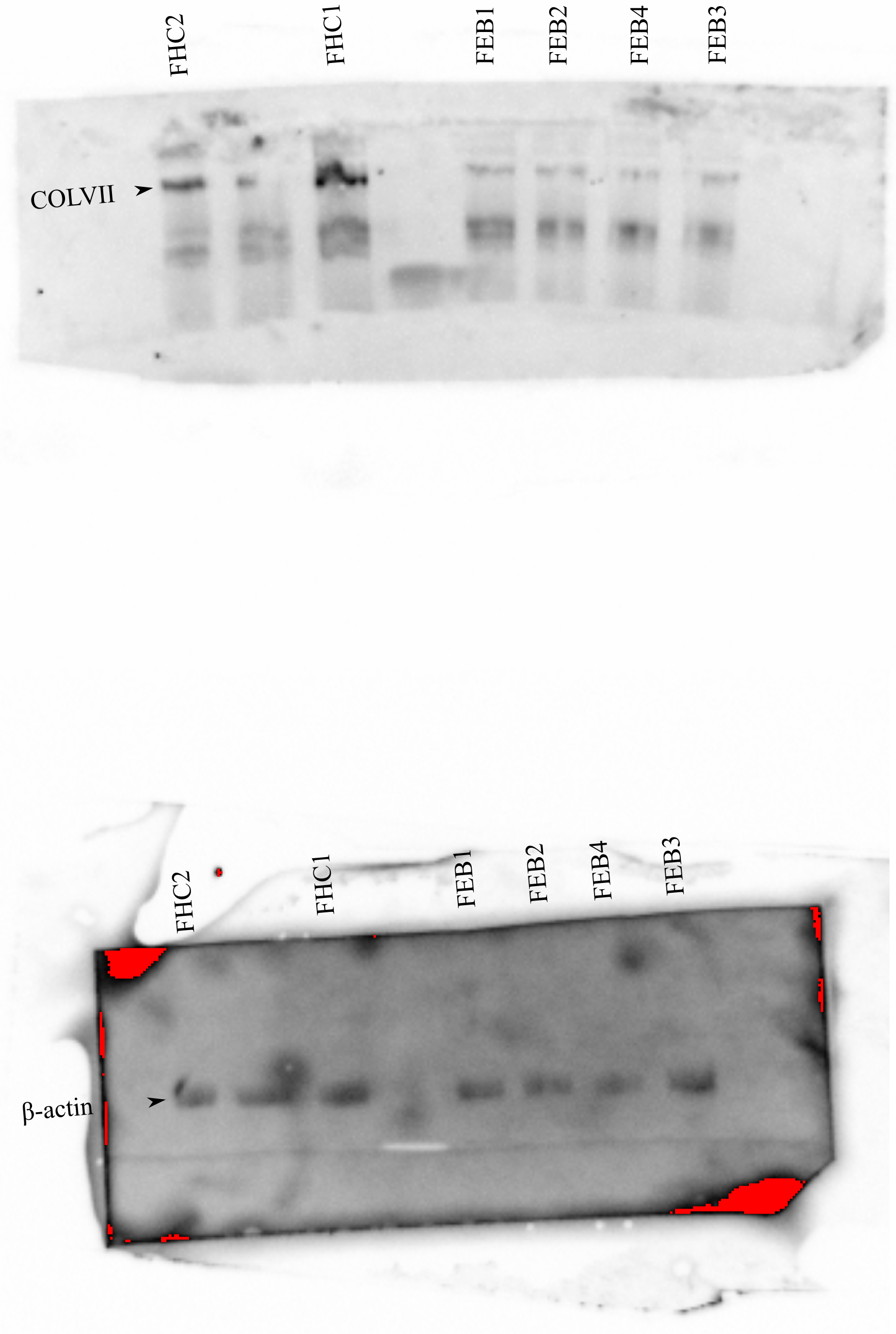

Supplement: Supplementary file 1 [file ijms-22-01792-s001.zip › ijms-1080037-proofed-supplementary/Figure S3.png]

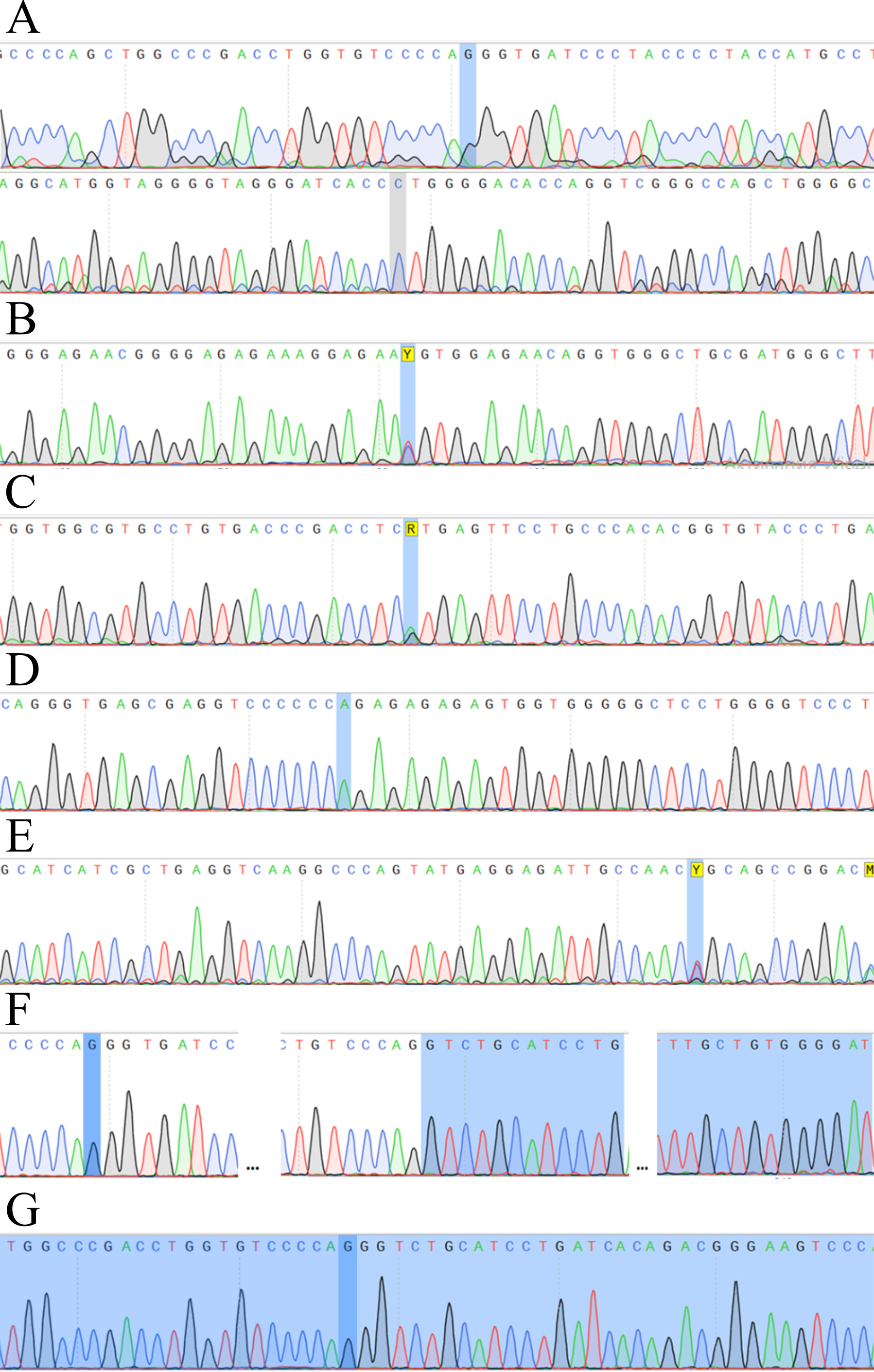

Supplement: Supplementary file 1 [file ijms-22-01792-s001.zip › ijms-1080037-proofed-supplementary/Figure S4.png]

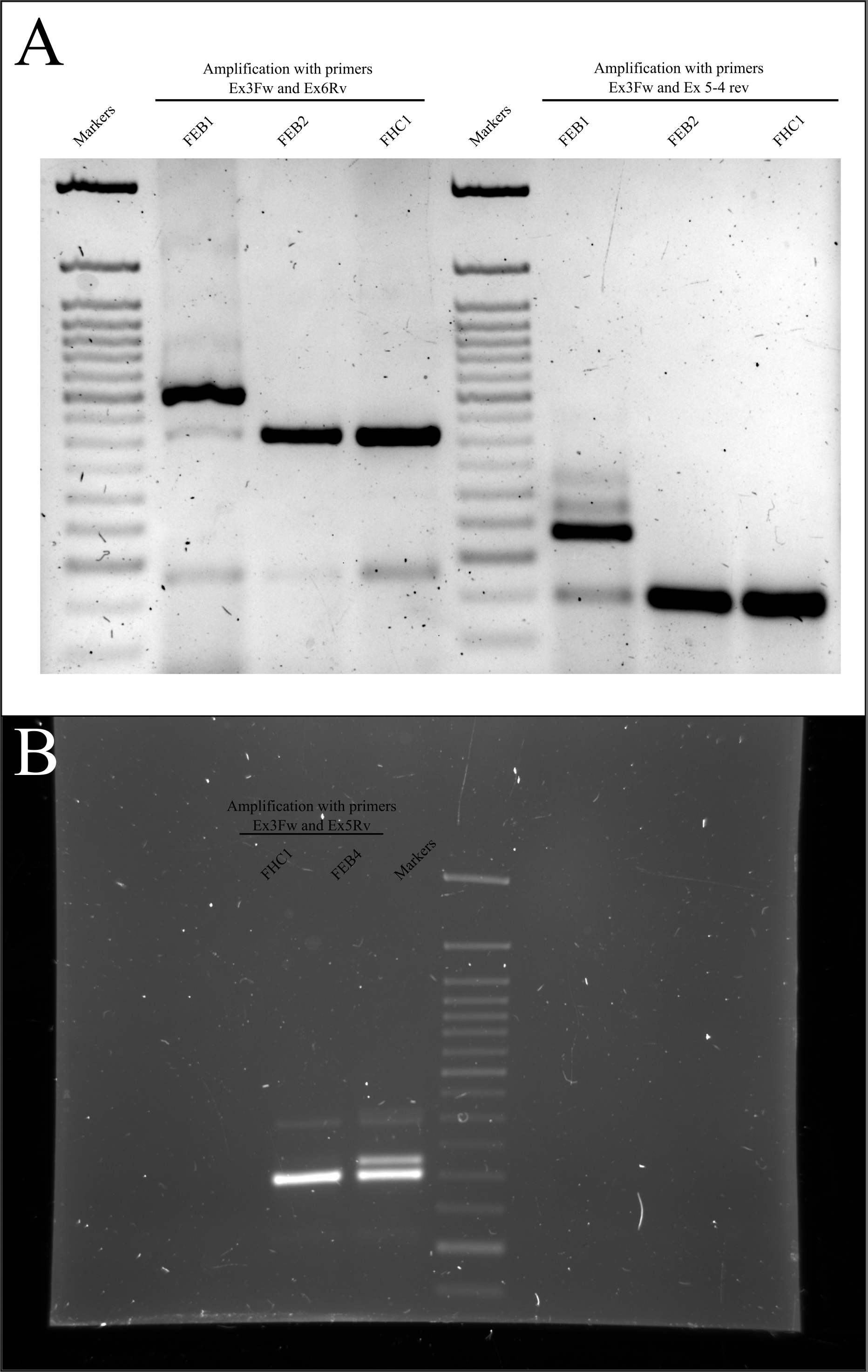

Supplement: Supplementary file 1 [file ijms-22-01792-s001.zip › ijms-1080037-proofed-supplementary/Figure S5.png]

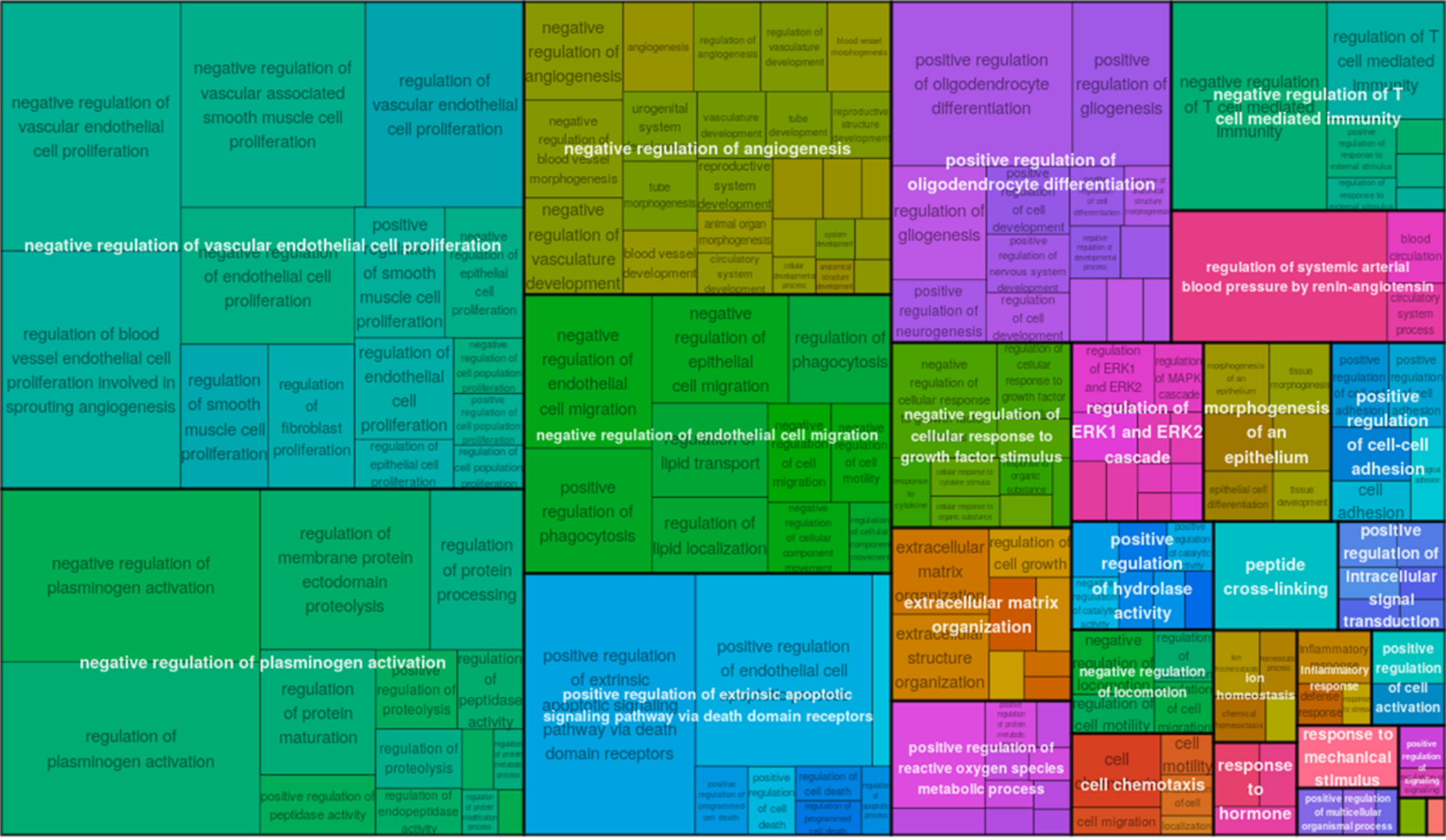

Supplement: Supplementary file 1 [file ijms-22-01792-s001.zip › ijms-1080037-proofed-supplementary/Figure S6 A.png]

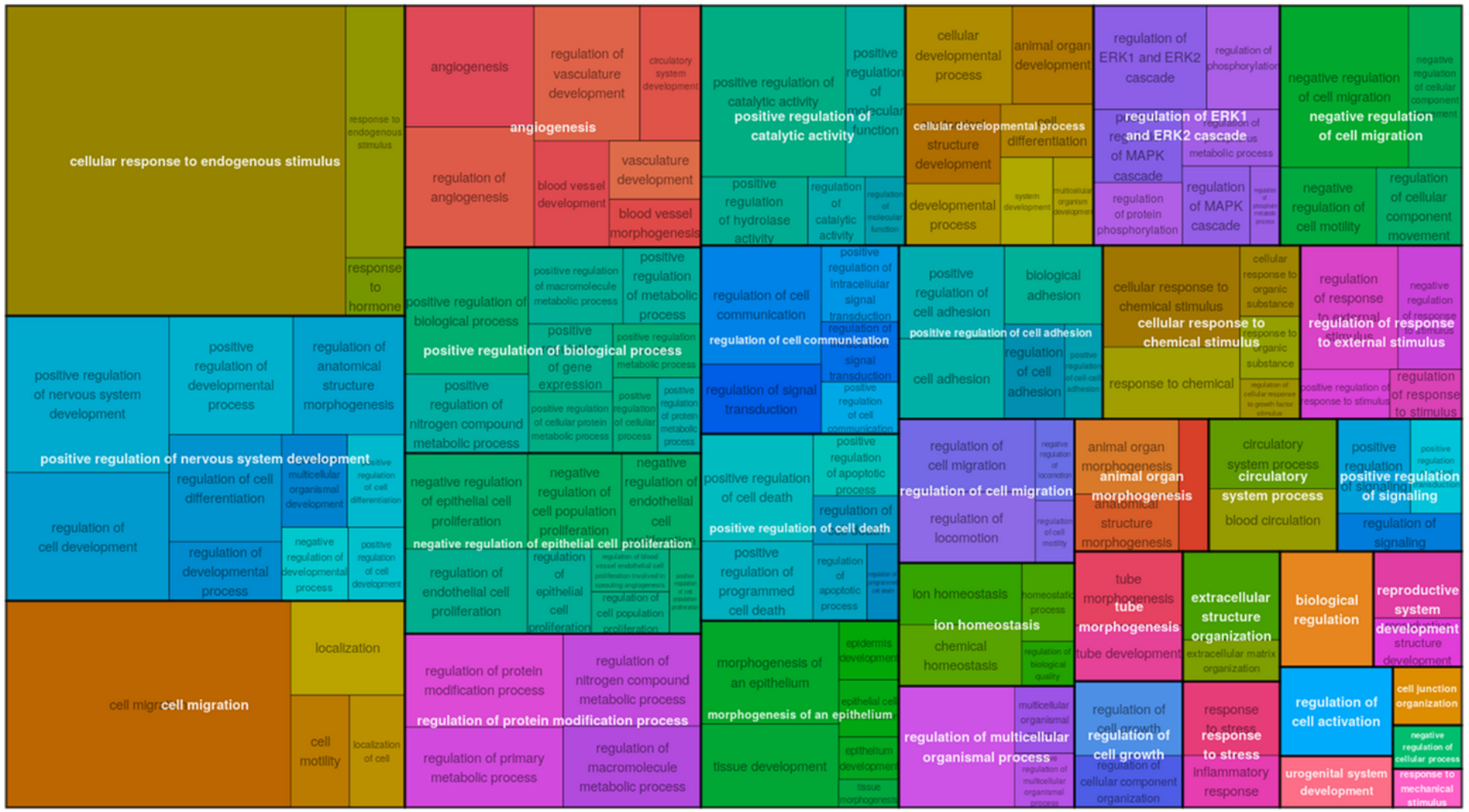

Supplement: Supplementary file 1 [file ijms-22-01792-s001.zip › ijms-1080037-proofed-supplementary/Figure S6 B.png]

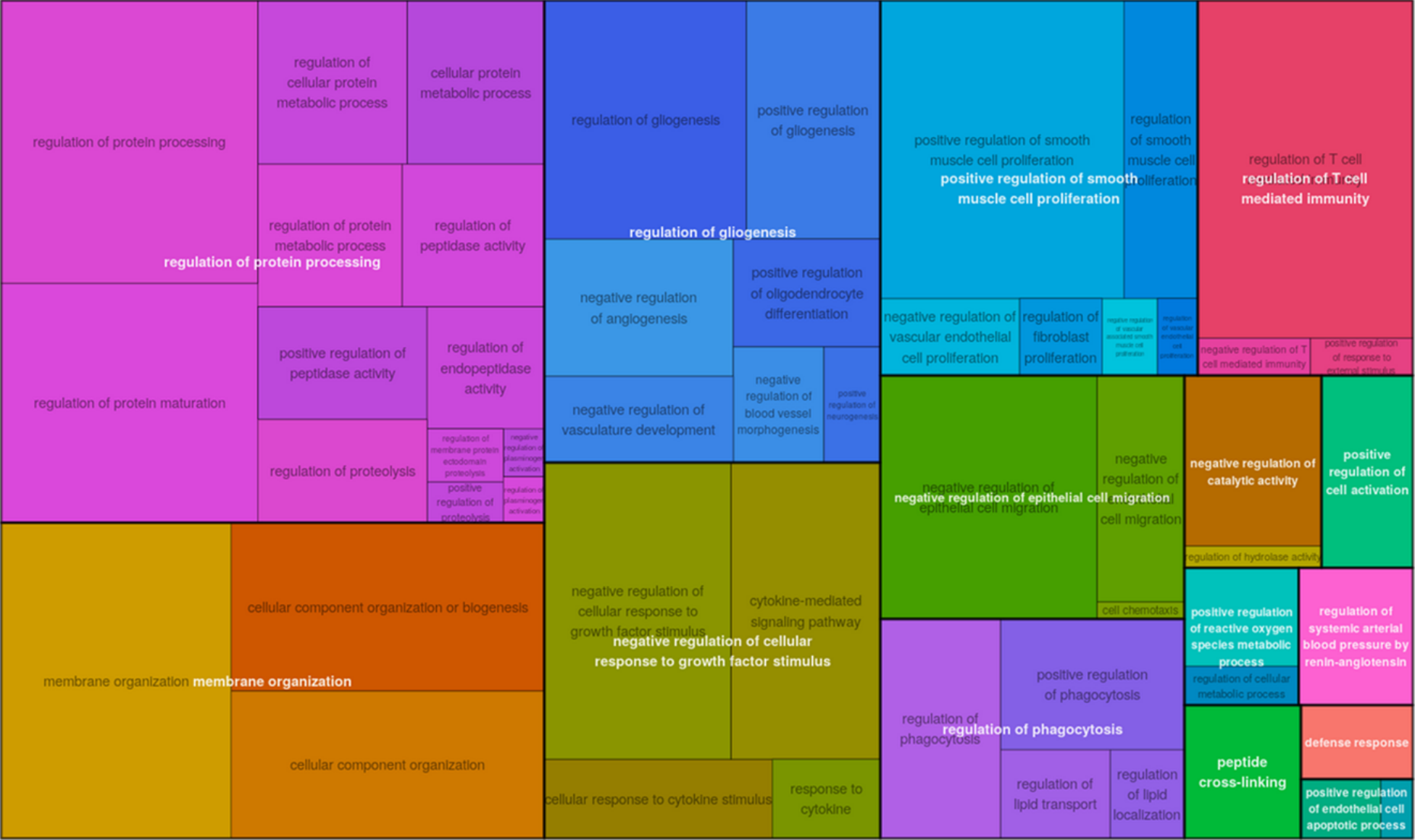

Supplement: Supplementary file 1 [file ijms-22-01792-s001.zip › ijms-1080037-proofed-supplementary/Figure S6 C.png]

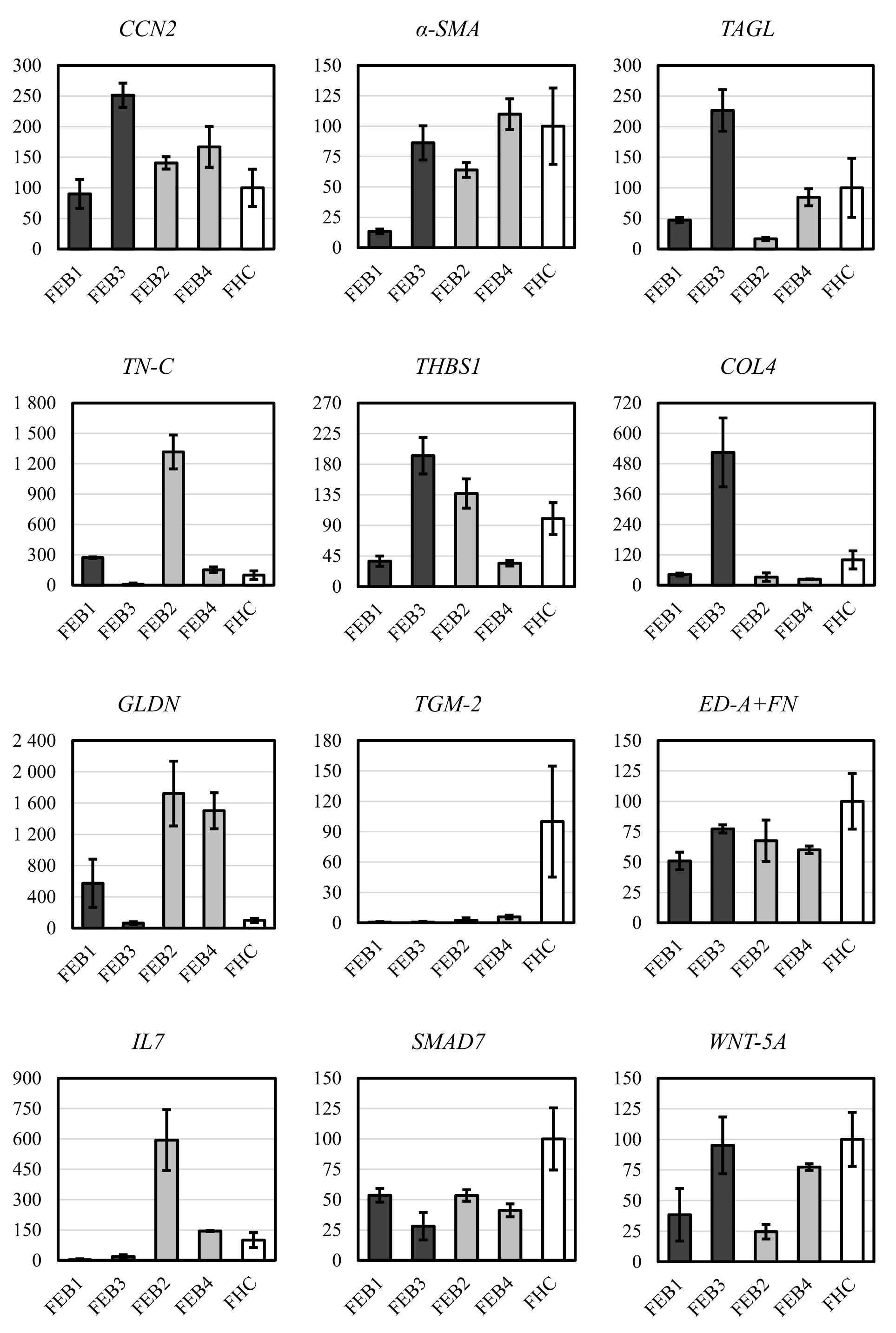

Supplement: Supplementary file 1 [file ijms-22-01792-s001.zip › ijms-1080037-proofed-supplementary/Figure S7.png]
